# What Radiation Oncology Wants Medical Oncology to Know

**DOI:** 10.6004/jadpro.2016.7.3.12

**Published:** 2016-04-01

**Authors:** J. Nicholas Lukens, Erin McMenamin

**Affiliations:** University of Pennsylvania School of Medicine, Philadelphia, Pennsylvania, and Hospital of the University of Pennsylvania, Philadelphia, Pennsylvania

Optimal radiation treatment planning and symptom management for radiotherapy toxicities are keys to patient adherence and good outcomes, according to radiation oncology specialists who described the field at JADPRO Live.

J. Nicholas Lukens, MD, of the University of Pennsylvania School of Medicine, Philadelphia, Pennsylvania, described the basics of radiation oncology, indicating that its target is cellular DNA.

"What we are usually trying to do with radiation is to cause double-strand breaks in cancer cells," he said. "Cancer cells divide faster than normal cells but they are not good at repairing DNA and therefore are more vulnerable to radiotherapy."

Focused radiotherapy stops tumor cells from dividing, thus controlling tumor growth, and it ideally kills them and cures the cancer, Dr. Lukens said.

## MAIN FORMS OF RADIATION THERAPY

External beam radiation therapy (EBRT), also known as 3D conformal radiation, includes intensity-modulated radiation therapy (IMRT) and stereotactic body radiotherapy (SBRT). The other form is brachytherapy (interstitial or intracavitary), where a radiation source is placed within or directly adjacent to the tumor.

One of the greatest advances in the field is the ability to "shape" the beam of radiation to conform to the tumor. This is accomplished by carefully measuring tumor volume and other parameters using PET/CT. A highly individualized treatment plan is developed from this information, which is designed to deliver tumoricidal doses to tumors while sparing normal tissue.

## PREPARING PATIENTS FOR RADIOTHERAPY

Erin McMenamin, MSN, CRNP, AOCN®, ACHPN, of the Hospital of the University of Pennsylvania, Philadelphia, Pennsylvania, discussed the steps in initiating treatment.

Patients often wonder, she said, why they don’t start radiation therapy right away, but the execution of the personalized treatment plan takes time. "It’s a precise treatment based on the anatomy of the patient," she emphasized.

A scan of the tumor site is performed, and "contouring" is done from the picture it generates. "The physician goes slice by slice from the scan to outline where the radiation should be delivered," she said.

"Then a radiation plan is generated. How the radiation will be administered is figured out by a dosimetrist. This is where the beams go in, at what angles, how to converge the beams to hit the target and spare normal tissues," she explained.

A physicist evaluates the plan, which is further peer-reviewed and modified as needed. In the case of radiotherapy for head and neck cancer, a mask is created that aligns treatment and gives only 1 to 2 mm of leeway. Patients are secured on the table, provided with music of their choice and given antianxiety medication if necessary.

Advanced practitioners can guide these patients through this challenging process, she said.

## TISSUE TOLERANCE AND TOXICITY

Tissue tolerance to radiation is site-specific. The acute side effects are a result of damage to the "early-responding" tissue, while late side effects come from damage to "late-responding" tissue.

Acute side effects of radiation therapy are often observed in the skin (erythema, desquamation), oral mucosa (mucositis), and small bowel (diarrhea)—areas with rapidly dividing cell populations that slough off the mucosal surface and are not replaced soon. Acute side effects occur at a predictable, fixed time point from the start of radiotherapy.

Late side effects occur in areas in which cells do not rapidly divide, such as the spinal cord (myelitis), heart (pericarditis), lung (pneumonitis, fibrosis), kidney, and liver.

"Conventional wisdom is that we shouldn’t give the whole radiation dose in one shot, so we fractionate," Dr. Lukens continued. "It’s not possible to give patients a single dose large enough to sterilize a tumor without also leading to a major risk of normal tissue injury. Multiple small doses of radiation make treatment safer."

## FRACTIONATION

Radiation is therefore delivered in fractions. With hypofractionated radiotherapy, the total dose is divided into large doses and treatment is given once a day or less often. With hyperfractionated radiotherapy, the total dose is divided into small doses and treatments are given more than once a day.

"When delivering radiotherapy to large volumes that are limited by normal tissue tolerance, you can get away with higher total doses if you fractionate, since fractionation allows time for normal tissues to recover," he explained. 

Palliative regimens tend to use hypofractionation (SBRT), i.e., radiation delivered in fewer fractions; it also works well in slow-growing tumors, which tend to be less sensitive to radiation. Curative treatment is often hyperfractionated, especially for tumors with rapidly diving cells.

"With increasingly conformal means of delivering radiation, we are seeing increasing use of hypofractionation," Dr. Lukens said. "This is accompanied by the potential for an increase in late side effects that won’t be readily apparent (such as vertebral body collapse, which was recently documented), and we will need to monitor for this."

## IMRT USES

With IMRT, radiation therapists use more angles to essentially "paint" the dose into the target. IMRT allows highly conformal dose distribution; can create sharp dose gradients that "curve" around a critical structure; and allows dose escalation for tumors directly adjacent to critical structures. However, IMRT also can increase gastrointestinal toxicity due to the large volume exposed, and potentially increase the risk of secondary malignancy.

RapidArc is a form of IMRT in which the machine rotates while shaping and delivering its dose. In the case of nasopharyngeal carcinoma, for example, it delivers tumoricidal doses into the tumors without exceeding 54 Gy to the brain stem, which would be disabling (Figure). "This is faster, and it’s a good option for patients who have discomfort on the table," he said.

**Figure:**
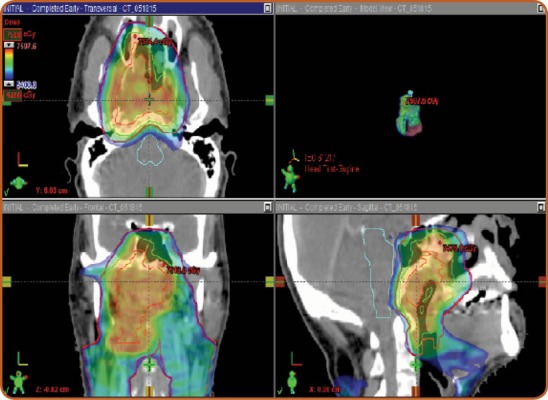
RapidArc for nasopharyngeal carcinoma invading the base of the skull. From the personal archive of Dr. Lukens.

The use of protons further spares critical normal tissue, as a lower radiation dose is delivered proximal to the tumor. Pencil-beam protons, which are fine beams of protons steered by a magnet, are an excellent treatment for complex target volumes.

## DEVELOPMENT OF ACUTE AND LONG-TERM TOXICITIES

Ms. McMenamin reiterated that radiation above the level of tissue tolerance increases the potential for higher grade acute and long-term toxicities. This includes greater risk for skin toxicity, pain, hemorrhage, and necrosis as acute effects, and the development of fistulas, fibrosis, and strictures as late effects.

Noting that patients attribute most any physical complaint after treatment to radiation exposure, she emphasized that radiation therapy side effects are limited to the area of the body where the radiation dose is delivered.

"I worked in palliative care before radiation oncology, and everything was blamed on opioids. Now everything is blamed on radiation," Ms. McMenamin said.

Patients also assume that radiation lingers in the body. They often ask how long it takes "for the radiation to get out of my system," she said. Radiation does not persist. The only patients who are "radioactive" are those who have had radioactive substances ingested (ie, radioactive iodine) or injected (ie, brachytherapy). These substances do remain in the body for some time, and radiation precautions are required during this period.

## HEAD AND NECK CANCER: COMMON SIDE EFFECTS

Ms. McMenamin helps manage patients with head and neck cancer. She described the following toxicities in this group as being dermatitis, dysgeusia, dysphagia, fatigue, lymphedema, odynophagia, weight loss, and xerostomia (both dry mouth and over-production of thick saliva). 

She noted that fatigue is very common and can be ameliorated through exercise and maintenance of a stable weight; weight maintenance is also critical for the correct fit of the mask worn during therapy. Lymphedema is seen more commonly when radiotherapy is delivered after neck dissection. Pain with swallowing is common, and can sometimes be prevented with a neuroactive agent such as gabapentin.

Dermatitis is not a result of "radiation burns," but by a number of processes: immediate structural tissue damage, generation of short-lived free radicals, irreversible double-stranded DNA breaks, and an inflammatory response in the epidermis and dermis.

"Daily treatments do not allow time for cells to repair tissue or DNA damage, leading to erythema," she explained.

Patients treated with protons may have more skin reactions, including blisters. An astringent followed by a moisturizer is the recommended treatment.

Mucositis occurs when stem cells in the basal layer are sloughed off and there are no new cells to replace them, leading to ulceration. This happens 2 to 3 weeks after radiotherapy starts and peaks the week after it is completed.

"We pay close attention to symptoms and try to be proactive," she said. "We know that if you skip treatments, you reduce the ability to cure." By harnessing the strengths of her multidisciplinary team members, she said, "I think we have better outcomes."

